# The Intersection of the Pathogenic Processes Underlying Psoriasis and the Comorbid Condition of Obesity

**DOI:** 10.3390/life14060733

**Published:** 2024-06-07

**Authors:** Emanuele Scala, Laura Mercurio, Cristina Albanesi, Stefania Madonna

**Affiliations:** Laboratory of Experimental Immunology, Istituto Dermopatico dell′Immacolata-Istituto di Ricovero e Cura a Carattere Scientifico (IDI-IRCCS), 00167 Rome, Italy; l.mercurio@idi.it (L.M.); c.albanesi@idi.it (C.A.); s.madonna@idi.it (S.M.)

**Keywords:** psoriasis, pathogenesis, obesity, biological therapy, molecular targets, adipokines

## Abstract

In the past decade, our understanding of psoriasis pathogenesis has made significant steps forward, leading to the development of multiple game-changing therapies. While psoriasis primarily affects the skin, it is increasingly recognized as a systemic disease that can have effects beyond the skin. Obesity is associated with more severe forms of psoriasis and can potentially worsen the systemic inflammation and metabolic dysfunction seen in psoriatic patients. The exact mechanisms underlying the link between these two conditions are not fully understood, but it is believed that chronic inflammation and immune dysregulation play a role. In this review, we examine the existing body of knowledge regarding the intersection of pathogenic processes responsible for psoriasis and obesity. The ability of biological therapies to reduce systemic and obesity-related inflammation in patients with psoriasis will be also discussed.

## 1. Introduction

Psoriasis and obesity are prevalent chronic conditions that globally affect people of all ages [1,2,3]. Individuals with psoriasis are more susceptible to developing obesity due to factors such as reduced physical activity, unhealthy dietary habits, and psychological distress caused by visible skin lesions [4]. Moreover, psoriasis produces a wide range of pro-inflammatory mediators not only in the lesions but also in the circulation, causing systemic inflammation and impairment in the adipose immune system. On the other hand, obesity can lead to inflammation, which can trigger further psoriasis [5,6]. So far, treating obese patients with psoriasis has been challenging, as they may have a poorer response to systemic treatments [6]. In this article, we provide an overview of the current understanding of how psoriasis and obesity influence each other and the impact that biological therapy has on psoriatic patients with the condition of obesity.

## 2. Materials and Methods

This is a narrative review. The PubMed database was consulted, from its inception until March 2024, using the search term “psoriasis” [all fields]; the keywords “obesity”, “cytokines”, or “adipokines”; and “AND” as operator. A total of 62 papers reporting the search terms in the title were selected. The studies were explored and documented with additional literature data. Overall, 140 articles were included in the body of this review. The selection of the articles was performed independently by E.S., L.M., C.A., and S.M. All the articles were reviewed by C.A. and S.M., who also made the final decision in the case of a disagreement between the initial selectors.

## 3. Psoriasis

### 3.1. Epidemiology, Clinical Features, and Quality of Life

Psoriasis is a non-communicable inflammatory skin disease that affects approximately 2–3% of the population worldwide [1]. It can occur at any age but most commonly develops between the ages of 15 and 35 [2]. Both genetic and environmental factors are thought to contribute to the development of psoriasis [7,8,9,10]. The human leukocyte antigen *(HLA)-Cw6* is the disease allele conferring the greatest risk of psoriasis [7]. Other genes, such as *IL12B*, *IL23R*, and *CARD14*, have also been linked to it [8,9]. Clinically, psoriasis is characterized by the presence of red, scaly patches on the skin, accompanied by intense itching and inflammation. It is common at the extensor sites of the extremities and the umbilicus and may present as the inverse phenotype in the axillary and inguinal regions. The disease severity varies from mild to severe, and its symptoms may come and go in flare-ups. It can have a significant impact on a person’s quality of life, both physically and emotionally [11]. The visible nature of the condition can lead to feelings of self-consciousness, embarrassment, and low self-esteem. People with psoriasis may report feeling stigmatized and judged, leading to social isolation and decreased participation in daily activities [12]. Its negative impact on social relationships, work, and intimate experiences can further exacerbate feelings of depression, anxiety, and stress [11,12].

### 3.2. Pathogenesis

Psoriasis manifests in predisposed individuals due to aberrantly mounting skin immune responses triggered by specific environmental factors [13,14]. These factors include physical trauma and bacterial or viral infections that activate innate immune responses by stimulating the formation and release of nucleic acid/autoantigen complexes by the damaged skin cells. For example, complexes formed by cathelicidin LL37 and self-DNA/RNA fragments activate plasmacytoid dendritic cells (pDCs), a subset of DCs that release high levels of interferon (IFN)-α and tumor necrosis factor (TNF)-α [15].

The recruitment of pDCs to psoriatic skin is facilitated by the chemokine chemerin, along with other innate immune cells such as neutrophils, macrophages, monocytes, and mast cells in the dermis [16]. The local production of IFN-α and other type I IFNs triggers keratinocyte immune activation and myeloid DC (mDC) maturation, initiating the adaptive immune response phase. As a result, an inflammatory environment rich in interleukin (IL)-23/IL-17 cytokines is established in psoriatic skin, with DC- and macrophage-derived IL-23 promoting type 17 helper (Th17) and cytotoxic (Tc17) effector responses [17,18]. In parallel, mDCs induce the IL-12/IFN-γ cytokine axis, accountable for an IFN-γ-dependent transcriptomic signature and the prevalence of Th1 and Tc1 cells in psoriasis plaque lesions [19,20].

Innate lymphoid cells (ILCs) and γδ-T cells, alongside mast cells and neutrophils, also significantly contribute to psoriasis development by releasing substantial levels of IL-17 and IL-22 [21,22]. Following the extensive expansion of effector T cells and the massive production of IL-17 and IL-22 in psoriatic skin, epidermal hyperplasia and aberrant cornification processes occur [23]. IL-17 also plays a role in activating keratinocytes to produce chemokines, such as CXCL1/CXCL2/CXCL8 and CCL20, which, respectively, recruit neutrophils and T cells, as well as antimicrobial peptides, including members of the S100 family [24,25]. Therefore, IL-17 serves as a key component in the pathogenic pathways connecting T cells and keratinocytes.

In addition, T-cell-derived IFN-γ and TNF-α stimulate numerous inflammatory pathways in resident skin cells, with each cytokine influencing different responses and exhibiting a degree of synergism in modulating the gene expression in psoriasis lesions [24,25,26]. For instance, IFN-γ-induced effects are enhanced by TNF-α, and together they activate several transcription factors, such as STAT1, STAT3, and NF-κB, which cooperate to upregulate pathogenic genes. Among them, the ICAM-1 adhesion molecule on resident skin cells facilitates the attachment and migration of circulating leukocytes [24], as well as various chemokines targeting the immune cells and pro-inflammatory cytokines like IL-6 and IL-1, which further support the expansion of the Th17 cells. Importantly, analyses of the transcriptional profiles in psoriatic skin lesions have revealed that the IFN-γ signature predominates, even though IL-17 and TNF-α are potent inducers of a wide array of genes [24,25]. Additionally, studies have revealed the pivotal role of IL-22 in psoriasis pathogenesis, as it activates genes dependent on STAT3 involved in differentiation and proliferation processes. However, IL-22 induces a more limited set of genes compared to IL-17 in human psoriatic skin lesions [27]. Among these genes, the CXCL8 and CCL20 chemokines are abundantly induced by IL-22, along with the HBD-2, HBD-3, and S100 antimicrobial peptides.

Although T lymphocytes play a crucial role in initiating immune responses in psoriasis, intrinsic abnormalities in the keratinocytes leading to an abnormal reaction to lymphokines may also be significant in the condition. In fact, several allelic variants of genes controlling keratinocyte inflammatory activation, as well as proliferation or differentiation processes, can predispose patients to psoriasis [28]. Several SNPs were found in genes encoding molecules involved in IL-17 or TNF-α intracellular signaling (i.e., STAT3, NF-κB, and Act1) [28,29]. However, functional studies correlating genetic variants with keratinocyte susceptibility to inflammatory cytokines are lacking.

In the past, the research efforts that have contributed to identifying the pathogenic processes in psoriasis have provided valuable insights into its intricate nature, highlighting cellular and molecular protagonists as crucial therapeutic targets. Among them, IL-17A and IL-23 have emerged as pivotal pathogenic molecules in psoriasis, and immunomodulation with biologics aimed at targeting them has resulted in substantial improvement in the disease symptoms and quality of life for patients [30,31]. Although this progress is remarkable, a remains unknown, especially regarding prevention, for example, in the identification of pathogenic predictors of the condition of psoriasis and response to therapies.

## 4. Obesity

Obesity is a pathological condition characterized by an excessive accumulation of body fat, with multiple factors contributing to its prevalence and incidence worldwide [32,33]. These factors include genetic predisposition, dietary habits, and environmental influences. The World Health Organization (WHO) defines obesity as having a body mass index (BMI) ≥ 30 kg/m^2^ [33]. Both obesity and psoriasis are linked to an increased risk of cardiovascular disease, type 2 diabetes, metabolic syndrome, and fatty liver disease [32,33,34,35]. Therefore, their co-occurrence may further exacerbate the risk of these comorbidities.

### 4.1. The Relationship between Psoriasis and Obesity

Epidemiologically, a non-causal association between psoriasis and obesity has been described. A population-based study in 2006 found that the prevalence of obesity in psoriatic patients was 20.7%. In particular, those with severe psoriasis had higher adjusted odds of obesity (OR, 1.79; 95% CI, 1.55–2.05) [36]. Subsequent studies in France reported an even higher prevalence, of up to 25%, in patients with psoriasis [37]. An extensive meta-analysis showed that psoriatic patients have a more than 50% increased risk of obesity compared to the general population [38]. Research conducted by Snekvik et al. evaluated the effect of BMI, waist circumference, waist–hip ratio, and 10-year weight change on the onset of psoriasis. The study revealed that individuals who were obese had a 1.87 times higher risk of developing psoriasis, and those who gained 10 kg or more experienced a 1.72 times greater risk compared to individuals who maintained a consistent weight [39]. Similar findings were observed in a prospective study of 121,700 female nurses, where obesity was associated with a 1.63 times higher risk of incident psoriasis [40]. The data support that being obese and experiencing long-term weight gain doubles the risk of incident psoriasis.

In addition to the epidemiological findings, studies have suggested a potential common genetic background between obesity and psoriasis [41,42,43,44]. A trans-ethnic Mendelian randomization study provided evidence of a causal link between obesity and the risk of psoriasis in European and Japanese populations [41]. Additionally, obesity is more frequent in patients with psoriatic arthritis (PsA) than in those with other inflammatory types of arthritis such as rheumatoid arthritis [45]. In a cohort study involving 89,049 participants (included from 1991 to 2005), a higher BMI was associated with an increased risk of incident PsA [46]. Furthermore, lipid metabolism in psoriasis may be influenced by epigenetic mechanisms such as changes in gene expression induced by DNA methylation, modifications to histones, and the involvement of non-coding RNAs [43,44].

**Figure 1:**
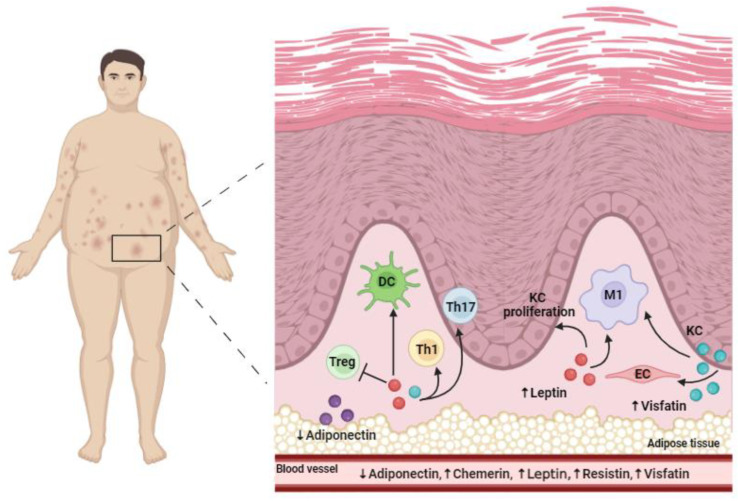
Simplified overview of the role of adipokines in chronic plaque psoriasis. Created using BioRender.com. Abbreviations: DC, dendritic cell; EC, endothelial cell; KC, keratinocyte; M1, macrophage 1 phenotype; Th, T helper cell; Treg, regulatory T cell.

However, the exact pathophysiological link between obesity and psoriasis is not fully understood, but it is hypothesized that increased systemic inflammation induced by adipose tissue, particularly visceral fat, may contribute to the development of chronic inflammation, exacerbating psoriasis [47,48]. Indeed, the adipose tissue, in addition to adipocytes, contains endothelial cells, fibroblasts, macrophages, myeloid cells, and T cells [48]. In a lean state, Th2 cells and Treg, Breg, and invariant NKT cells are the dominant immune cell populations, whereas increased adiposity positively correlates with a marked infiltration of pro-inflammatory cells, including Th1 and Th17 cells [48]. This infiltration causes an enhanced expression of inflammatory cytokines that are central to psoriasis pathogenesis, such as IL-17, IL-22, and IFN-γ. The potential role of obesity as an aggravating factor that contributes to psoriasis severity found confirmation in an obese mice model demonstrating the exacerbation of psoriasis-like lesions after imiquimod induction that was associated with an enhanced expression of IL-17A, IL-22, IL-23p19, IL-17C, and β-defensin 3 [49]. Moreover, obesity may contribute to psoriasis through adipokine activity, which might have pro-inflammatory effects on both immune and skin tissue cells (Figure 1).

### 4.2. Adipokines

Adipokines, secreted by skin-resident and immune cells, constitute a heterogeneous group of proteins including adiponectin, chemerin, leptin, resistin, and visfatin [50,51].

Adiponectin, an anti-inflammatory adipokine with insulin-sensitizing and cardioprotective properties, is typically reduced in obese subjects [52]. Studies have shown that it can inhibit keratinocyte proliferation and the production of pro-inflammatory molecules like IL-6, CXCL8, and TNF-α [53,54]. Adiponectin also plays a role in modulating immune responses by affecting the activation and function of DCs and monocytes, as well as promoting the expression of M2-phenotype markers in human monocyte-derived macrophages [55,56]. Research by Surendar and colleagues revealed that in mice on a high-fat diet, adiponectin reduces the number of IFN-γ^+^ and IL-17^+^ CD4^+^ T cells and hinders the differentiation of naïve T cells into Th1 cells and Th17 cells [57]. In psoriasis, decreased levels of adiponectin in the blood have been observed, especially in patients with severe disease, and these levels are negatively correlated with blood levels of IL-6 and TNF-α [58,59]. Furthermore, the expression of adiponectin receptor (ADIPOR1) is reduced in the psoriatic epidermis compared to healthy skin [54].

Chemerin is recognized as a pro-inflammatory adipokine that was initially identified as a chemoattractant for pDCs, macrophages, and NK cells [16,60]. It has been linked to lipid metabolism, with higher serum levels found in obese people [61]. In human keratinocytes, the expression of chemerin is stimulated by cytokines commonly associated with the acute phase of psoriasis, such as oncostatin M and IL-1β [62]. In psoriatic skin, chemerin expression is specifically related to the early stages of disease development and pDC infiltration in the dermis [16]. Chemerin was shown to increase the secretion of inflammatory factors, activate NF-κB, and worsen psoriasis symptoms in a murine model of imiquimod-induced psoriasiform dermatitis [63]. Elevated levels of circulating chemerin have been observed in psoriatic patients compared to healthy subjects, with a positive correlation with obesity-related clinical parameters [64]. Patients with PsA have also been found to have increased levels of chemerin in their blood [65].

Leptin plays a role in regulating energy balance by decreasing appetite [66,67]. In obese individuals, there is a connection between leptin and decreased receptor function, leading to increased food intake. The levels of leptin in the blood are higher in obese subjects compared to lean ones [68]. Leptin also has inflammatory and immune-modulating effects. It stimulates the production of certain cytokines (e.g., IL-6, CXCL8, and TNF-α) in the keratinocytes [69] and promotes proliferation [54]. In a mouse study, leptin was observed to have similar effects on the skin [70,71]. It promotes Th1 immune responses and inhibits Th2 responses [72,73], as well as inducing the release of CXCL8, TNF-α, IL-1β, and IL-1ra from the monocytes [74] and IL-1β, IL-6, TNF-α, and IL-12 from the macrophages [75,76]. Moreover, Mattioli and colleagues demonstrated that the exposure of naïve T cells to leptin-treated DCs led to an increased in Th1 polarization [77]. However, the role of leptin in psoriasis pathogenesis remains unclear, as studies on the leptin levels in the blood and skin of psoriatic patients have yielded conflicting results. Johnston et al. found no significant differences in the serum leptin levels or the levels of leptin receptors between psoriatic and healthy individuals [74]. However, reduced levels of leptin receptor mRNA and protein were detected in psoriasis lesions compared to healthy and uninvolved psoriasis skin [74]. On the other hand, meta-analyses have shown that the circulating leptin levels are higher in individuals with psoriasis, correlating with disease severity [78].

Resistin is a pro-inflammatory adipokine that is mainly produced by adipose tissue. Its name originates from its connection to obesity and insulin resistance [79]. Studies have shown that elevated levels of resistin are present in obese mice and are linked to decreased insulin sensitivity [80]. The role of resistin in human insulin resistance is not well understood, as the research has produced conflicting results. Patients with psoriasis have been found to have higher plasma and serum levels of resistin when compared to healthy individuals [78,81].

Visfatin, also known as the extracellular form of the nicotinamide phosphoribosyltransferase enzyme (eNAMPT), is a pleiotropic adipokine with pro-inflammatory properties that plays a role in several metabolic and inflammatory diseases [82,83]. Elevated blood levels of visfatin have been linked to obesity and insulin resistance [84,85]. Visfatin expression is increased in psoriatic lesions, with in vitro studies showing that human keratinocytes and dermal fibroblasts release visfatin to promote endothelial cell proliferation, migration, and inflammation [86]. Visfatin has various effects on immune cells, including the expression of co-stimulatory molecules in monocytes and promoting T cell activation [87]. It also induces an inflammatory M1 phenotype in murine peritoneal macrophages and human monocyte-derived macrophages by activating STAT1/3 phosphorylation [88]. In addition, visfatin boosted the production of TNF-α-induced CXCL8, CXCL10, and CCL20 in human keratinocytes [89], as well as antimicrobial peptides such as CAMP, HBD-2, HBD-3, and S100A7 in a murine model of IMQ-induced psoriasis [90]. The levels of visfatin in the serum of psoriasis patients are significantly higher compared to those of healthy subjects and are correlated with the severity of the disease [91,92]. The exact molecular mechanism of visfatin’s action is still not fully understood. Research indicates that visfatin may bind to C-C chemokine receptor 5 (CCR5) in cancer cells as an antagonist [93] and promote inflammation by enhancing Toll-like receptor 4 (TLR4)-mediated pathways [94]. New theories suggest that visfatin may interact with other receptors, as its pro-inflammatory effects can occur independently of TLR4 [88].

## 5. Pharmacological Treatment of Psoriasis in Obese Patients

Generally, obesity is a negative predictor of the efficacy of both conventional and biological treatments, especially for those used at fixed dosage due to the altered pharmacokinetics and clearance of the drugs [95]. The therapeutic approach to psoriasis increasingly requires a comprehensive view, which reasonably considers beneficial effects not only for skin manifestations but also for psoriasis-related comorbidities.

### 5.1. Conventional Systemic Treatments

Traditional systemic therapies for psoriasis, including methotrexate, acitretin, cyclosporine, and phototherapy, do not affect the body weight of patients [48]. However, it is important to be aware that individuals with psoriasis, especially those who are obese, are more prone to liver steatosis and to consider the risk of liver toxicity when choosing a treatment. Regarding methotrexate mediation, it can be harmful to the liver, and acitretin should be used with caution in obese patients due to their increased risk of hypertriglyceridemia and hypercholesterolemia, particularly in those with diabetes or alcohol abuse. Cyclosporine is orally dosed according to the patient’s body weight (3–5 mg/kg daily), but obesity, as well as older age and hypertension, can increase the risk of nephrotoxicity induced by this drug. Lastly, photochemotherapy with psoralen plus UVA is unaffected by body weight since psoralen can be weight-adjusted. However, patients with abdominal obesity may be more susceptible to skin reactions and burns due to their closer proximity to the bulbs and consequently warrant more caution in the UV dose selection.

### 5.2. Non-Conventional Systemic Treatments

The biologics approved for the treatment of psoriasis can be classified as follows: (i) TNF-α inhibitors (infliximab, etanercept, and adalimumab); (ii) IL-12/23 inhibitors (ustekinumab); (iii) IL-17 inhibitors (ixekizumab, secukinumab, brodalumab); and (iv) IL-23 inhibitors (guselkumab, tildrakizumab, risankizumab) [96].

Infliximab, a chimeric monoclonal antibody, is dosed based on weight (5 mg/kg). However, a study in a real-life setting found that obesity can lead to a delayed response and lower overall efficacy [97]. Etanercept (a fusion protein consisting of the binding part of the human type II receptor for TNF-α-p75—linked to the Fc portion of IgG1) and adalimumab (human monoclonal antibody) are used at fixed doses, and their effects on obesity in patients with psoriasis are debated [98,99,100,101]. It has been suggested that etanercept could have neutral or even positive effects on weight management in these patients [98]. In contrast, other studies demonstrated that psoriatic patients who receive anti-TNF-α therapy may experience significant weight gain after 6 months of treatment [99,100,101]. Specifically, ~25% of patients treated with etanercept or infliximab exhibited a relevant increase in body weight ranging from 4 to 10 kg [100]. Physiologically, TNF-α stimulates adipocyte leptin synthesis in the adipocytes, induces lipolysis, and inhibits both lipogenesis and anabolic insulin-like growth factor 1 production [50,102,103]. Thus, TNF-α is thought of as an anti-obesity cytokine, limiting body mass increase. Thereby, the enhanced TNF-α levels detected in obese subjects are supposed to play a protective function. Conversely, TNF-α may also contribute to obesity, countering insulin receptor activity and inhibiting glucose transporter 4, with a consequent enhancement of insulin levels, which stimulates the hunger center [48]. So far, the mechanism underlying weight gain among psoriasis patients receiving biologics that target TNF-α remains unclear. Anti-TNF-α therapies in psoriatic patients do not significantly affect their serum concentrations of either leptin or resistin [104], although they were more likely associated with an increased body weight, which correlates positively with leptin serum levels, and may lead to a reduction in insulin resistance. Finally, anti-TNF-α treatments may indirectly have a positive effect on lean mass by increasing appetite [105].

Ustekinumab is a fully human monoclonal antibody targeting the IL-12 and IL-23 cytokines. It is dosed based on weight, with a 45 mg dose prescribed for patients weighing less than 100 kg, doubled for patients weighing over 100 kg. Hence, infliximab and ustekinumab could be the most suitable treatment options for obese individuals with psoriasis, as they are dosed according to body weight [106]. A study conducted in Italy found that ustekinumab does not lead to an increase in BMI in patients with chronic plaque psoriasis, unlike anti-TNF-α inhibitors [107].

Regarding IL-17 inhibitors, ixekizumab—a humanized monoclonal antibody targeting IL-17A—has shown efficacy in treating moderate to severe psoriasis even in individuals with obesity [108]. Secukinumab, the first fully human monoclonal antibody targeting IL-17A, demonstrated higher response rates in patients weighing less than 90 kg compared to those weighing 90 kg or more in a phase II study [109]. However, obesity tended to be a negative predictive factor of a “super-response” to the anti-IL-17 receptor A monoclonal antibody brodalumab over a period of 104 weeks of treatment in plaque psoriasis patients [110]. Yet research has indicated that blocking IL-17 could reduce systemic inflammation in psoriasis, potentially improving obesity-related inflammation as well [111]. Indeed, anti-IL-17 therapy could enhance metabolic factors such as insulin sensitivity and lipid profiles, ultimately improving obesity and the management of psoriatic patients. Although there are limited data on the effects of anti-IL-17 biologics on weight changes in psoriasis, some clinical trials and observational studies have indicated changes in weight as a secondary outcome [112,113,114]. However, there is controversary surrounding these results, as some studies demonstrate weight gain, weight loss, or no significant change in weight. For example, in a post hoc analysis of the FIXTURE-ERASURE-SCULPTURE studies, including 3010 patients who were treated with secukinumab, etanercept, or a placebo and followed up for 52 weeks, secukinumab was shown to reduce their BMI values but had no effects on plasma glucose, lipid, and liver enzyme parameters [112]. Conversely, in a retrospective study, Wang et al. examined the effect of secukinumab on 99 psoriatic patients during a 24-week treatment period and found their body weight and BMI were significantly increased at week 24 [113]. Additionally, in the UNCOVER studies, no significant changes were observed in body weight or serum lipid and glucose parameters after 60 weeks [114]. However, effectively treating psoriasis with anti-IL-17 biologics may result in improvements in quality of life and mental health, indirectly influencing lifestyle factors such as diet and exercise habits. These changes could impact weight management in psoriasis patients with obesity. Long-term data on the effects of anti-IL-17 biologics on obesity in psoriasis patients are limited, as most studies have relatively short follow-up periods. Further research is needed to understand the sustained effects of these medications on weight and metabolic health.

At present, limited research is available on the impact of IL-23 inhibitors on body weight. A recent multicenter retrospective study found that being overweight or obese did not affect the success of treatment with anti-IL-23 drugs in patients with psoriasis [115]. Specifically, guselkumab, a fully human IgG1λ monoclonal antibody targeting the p19 subunit of IL-23, has shown promising results in clinical trials. A subanalysis derived from head-to-head phase III trials (Voyage 1 and 2) testing guselkumab vs. adalimumab and a placebo showed sustained its superior efficacy compared to adalimumab and the placebo across all body weight classes [116,117]. Moreover, guselkumab is not reported to increase body weight or to alter lipid or glucose metabolism [48]. In sum, the effect of biological therapy on body weight and its efficacy in obese patients with psoriasis are depicted in Table 1.

Besides biologics, it is important to mention apremilast, an oral small-molecule phosphodiesterase (PDE)-4 inhibitor. This medication functions intracellularly by preventing the breakdown of cyclic adenosine 3′,5′-monophosphate, leading to higher levels of this molecule in PDE-4-expressing cells. Apremilast regulates the production of both pro-inflammatory and anti-inflammatory mediators such as IL-17, TNF-α, IL-23, IL-10, and TGF-β [118]. Inhibiting PDE-4 has been shown to decrease body weight by enhancing energy expenditure, improving glucose metabolism, enhancing metformin activity, and regulating adipose tissue inflammation [119,120,121,122,123,124]. Therefore, apremilast could be considered a viable treatment option for psoriasis in obese subjects.

### 5.3. Adipokines as Possible Markers of Response to Biologics

Circulating adipokine concentrations are altered in psoriatic patients and may play a role in the connection between psoriatic lesions and metabolic changes [125,126,127]. Leptin and resistin promote pro-inflammatory mediators (e.g., TNF-α and CXCL8) involved in psoriasis [128], while adiponectin can inhibit TNF-α [129]. Studies on the effects of biological therapy on these adipokines in psoriatic patients have shown inconsistent results [65,104,130,131,132,133,134,135,136]. Treatment with certain drugs (adalimumab, etanercept, infliximab, or ustekinumab) can reduce resistin concentrations, potentially impacting clinical outcomes [78]. We reported that visfatin expression significantly decreased after treatment with secukinumab, suggesting its potential as a biomarker for monitoring the therapeutic response in psoriasis [86]. Further research is needed to explore its predictive role in psoriatic patients, especially those with obesity-related comorbidities.

### 5.4. Management of Obesity and the Influence on Psoriasis

Obesity can have a significant impact on psoriasis progression. Weight loss and adopting healthier lifestyle habits, like a nutritious diet and increased physical activity, could improve psoriasis symptoms and reduce their severity. Certain medications used for weight management, such as metformin, liraglutide, and orlistat, have anti-inflammatory properties that may be beneficial for individuals with psoriasis [137,138,139].

Metformin is a medication used to treat type 2 diabetes in patients who have not been successfully treated with diet and exercise interventions alone. It is often used in overweight patients and can be taken alone or in combination with other diabetes treatments. Metformin helps to reduce high blood sugar levels, prevent inflammation, and normalize lipid and carbohydrate metabolism. It has also been found to be effective in treating psoriasis and metabolic syndrome when used in combination with other medications [137].

Liraglutide is a glucagon-like peptide-1 receptor agonist and a recommended supplement for managing weight in adults with obesity or overweight and weight-related co-morbidities. A case study showed an improvement in psoriasis before weight loss, suggesting the potential anti-inflammatory effect of liraglutide. However, it should be discontinued after 12 weeks if weight loss goals are not met [138]. Further randomized trials are needed to determine its effectiveness for psoriasis treatment.

Orlistat is a pancreatic lipase inhibitor recommended for weight loss in adults aged ≥18 with a BMI ≥ 28. By blocking the absorption of fats from the diet, orlistat helps in reducing overall caloric intake. If patients do not achieve at least a 5% reduction in their initial body weight after 12 weeks of treatment, it should be stopped. So far, there are no studies that have analyzed the effect of orlistat in obese subjects with psoriasis.

## 6. Conclusions and Future Perspectives

Psoriasis is a chronic inflammatory skin disease commonly linked to obesity, which can affect the severity of the disease and treatment outcomes. A holistic approach is necessary for treating these patients, encompassing lifestyle changes related to diet and exercise alongside medication. It is crucial that pharmacological treatments are supervised by healthcare professionals to ensure they are customized to individual needs and monitored for potential side effects.

We firmly believe that exploring the relationship between adipokines and inflammatory pathways in psoriasis can provide valuable insights and personalized treatment options. This research could not only advance our understanding of psoriasis but also other immune-mediated skin conditions associated with obesity. In the future, our goal is to investigate the involvement of adipokines in hidradenitis suppurativa [140], a chronic inflammatory skin condition associated with obesity that remains poorly understood.

## Figures and Tables

**Table 1:** Impact of biological therapy on obesity and body weight in psoriasis.

Biologics	Efficacy in Obese Patients	Effect on Body Weight
Anti-TNF-α	Potential decrease in efficacy	Could lead to weight gain
Anti-IL-12/23	Effective	Neutral influence
Anti-IL-17	Effective	No significant effects
Anti-IL-23	Effective	Data availability is limited

Abbreviations: IL, interleukin; TNF-α, tumor necrosis factor alpha.

## Data Availability

Not applicable.
